# Vibration-Based Fatigue Analysis of Octet-Truss Lattice Infill Blades for Utilization in Turbine Rotors

**DOI:** 10.3390/ma15144888

**Published:** 2022-07-14

**Authors:** Sajjad Hussain, Wan Aizon W. Ghopa, S. S. K. Singh, Abdul Hadi Azman, Shahrum Abdullah, Zambri Harun, Hawa Hishamuddin

**Affiliations:** Department of Mechanical and Manufacturing Engineering, Universiti Kebangsaan Malaysia, Bangi 43600, Malaysia; waizon@ukm.edu.my (W.A.W.G.); salvinder@ukm.edu.my (S.S.K.S.); hadi.azman@ukm.edu.my (A.H.A.); shahrum@ukm.edu.my (S.A.); zambri@ukm.edu.my (Z.H.); hawa7@ukm.edu.my (H.H.)

**Keywords:** vibration fatigue analysis, turbine rotor blades, octet-truss lattice, modal analysis, durability

## Abstract

Vibration fatigue characteristics are critical for rotating machinery components such as turbine rotor blades. Lattice structures are gaining popularity in engineering applications due to their unique ability to reduce weight and improve the mechanical properties. This study is an experimental investigation of octet-truss lattice structure utilization in turbine rotor blades for weight reduction and to improve vibration fatigue characteristics. One completely solid and three lattice infilled blades with variable strut thickness were manufactured via additive manufacturing. Both free and forced experimental vibration analyses were performed on the blades to investigate their modal and vibration fatigue characteristics. The blades were subjected to random vibration using a vibration shaker. The response was measured using a triaxial accelerometer in terms of vibration acceleration time histories in the X, Y, and Z directions. Results indicate a weight reduction of up to 24.91% and enhancement in the first natural frequency of up to 5.29% were achieved using lattice infilled blades. The fatigue life of the blades was investigated using three frequency domain approaches, namely, Lalanne, Dirlik and narrow band. The fatigue life results indicate that the 0.25 mm lattice blade exhibits the highest fatigue life, while the solid blade exhibits the lowest fatigue life of all four blades. The fatigue life of the 0.25 mm lattice blade was 1822-, 1802-, and 1819- fold higher compared to that of the solid blade, using the Lalanne, Dirlik, and narrow-band approaches, respectively. These results can serve as the first step towards the utilization of lattice structures in turbine blades, with thermal analysis as the next step. Therefore, apart from being light weight, the octet-truss lattice infilled blades exhibited superior vibration fatigue characteristics to vibration loads, thereby making them a potential replacement for solid blades in turbine rotors.

## 1. Introduction

Components of rotating machinery such as turbine blades are subjected to harsh environments with multiple types of loadings and high-frequency vibrations during their operational life. Thus, investigation of all the relevant parameters is mandatory for an efficient and durable design. This includes material properties, design features, vibration characteristics and durability [1]. For durability analysis, fatigue life is estimated at the design stage as well as during service life while considering the harsh operating conditions under which these blades operate [2,3]. These conditions include centrifugal loads due to rotation, vibration loads due to gas flow and thermal loads. Usually, the centrifugal loads cause the low cycle fatigue (LCF), while vibration loads result in high cycle fatigue (HCF) [4,5,6]. Sources of HCF damage in turbines include excitations due to flow perturbations; vibrations due to unbalance or misalignment; and aeromechanical instabilities [3]. The damage tolerance methods combined with probabilistic techniques are often used for the safe life design [7,8,9]. The structures subjected to cyclic loadings such as turbine blades are usually designed using probabilistic techniques. For this purpose, scatter plot of material properties against loads are considered to include the failure probability [10].

Due to the vital importance of turbine blades in turbine engines, considerable research has been carried out to develop various design and manufacturing optimization techniques to improve the performance efficiency and durability of turbine blades [11,12,13,14,15]. Weight reduction without compromising the desired characteristics and efficiency of turbine blades is a current popular area of research. Porous structures, formed by combination of three dimensional unit cells of various types with hollow space, known as lattice structures are utilized in various engineering applications for weight reduction [16,17,18,19,20]. Currently, the utilization of lattice structures in turbine blades is also being explored. Alkebsi et al. [21] proposed optimization of gradient lattice-based gas turbine blades using lattice structure topological optimization (LSTO) by taking stress into account. By using triply Periodic Minimal Surface (TPMS) lattice structures, a significant reduction in weight of the blades (up to 40%) and stress reduction (up to 52%) at operating conditions was achieved. Using a hybrid solid lattice structural optimization [22], a 14.8% increase in the first natural frequency and a 20.8% reduction in deformation was achieved for missile components. D Akzhigitov et al. [23] performed a parametric study on utilization of Kagome truss core in gas turbine rotor blades. They reported a reduction in stress level using topological optimization. S Hussain et al. [24] designed and manufactured octet-truss lattice-based gas turbine blades via additive manufacturing. They reported a weight reduction of up to 24.9%, a reduction in stress of up to 38.6%, and a reduction in deformation of up to 21.5% in lattice blades compared to that of solid blades.

For gas turbine blades, thermal loads and cooling effects are also critical and require profound investigations. Numerical techniques such as computational fluid dynamics (CFD) are employed for this purpose. Considerable research has been performed in this regard. For material selection, the combined effect of thermal and mechanical loads is considered to evaluate the performance of each type of blade material. Nickel-based superalloys are commonly used for gas turbine blades [25]; however, molybdenum-based alloys such as Mo–17.5Si–8B and Mo–9Si–8B exhibited low stress and strain levels compared to nickel-based superalloy CMSX-4 when subjected to thermomechanical loads, as reported by [26]. Turbine blades are internally cooled by the air spilled from compressor and sent into the internal channels to reduce the heat from the surface of the blades. Turbulence promoters inside the cooling channels are generally used to promote turbulence to improve the heat transfer efficiency. This results in unsteady, complex, and turbulent flow [27]. Since lattice structures are porous in nature, and octet-truss lattice structures have a complex shape, which can cause turbulence in air flow, octet-truss lattice structures can be helpful in heat transfer. Various experimental and numerical techniques, as well as models are employed to measure the cooling effectiveness such as in [28,29]. In order to improve the cooling effectiveness, various techniques are utilized such as impingement cooling [30] or thermal barrier coatings (TBC) [31].

Fatigue testing procedures with resonant conditions have been in practice for over a century. In vibration fatigue testing, the first step is to identify the natural frequencies and associated mode shapes. Based on the natural frequencies, the frequency range of the vibration shaker is adjusted to produce a desirable stress field. Modal parameters can be obtained (natural frequencies, mode shapes, and damping) by FEA; however, experimental validation must be carried out for accuracy of results [2]. Usually, for vibration fatigue analysis, the structural dynamics of the system, its response analysis, and fatigue analysis are required [32,33].

For the last fifty years, fatigue life estimation under random vibration loadings has been a popular active research area, especially for the last two decades. A number of novel methods have been proposed to analyze different loading conditions such as Gaussian and non-Gaussian, stationary uniaxial and multi-axial, with wide-band or narrow-band frequency content [34,35]. Despite all the improvements in theoretical and numerical investigation approaches developed over the years, experimental testing and investigation are still important steps to determine the accuracy of spectral methods. Moreover, full-scale durability tests that are close to the actual service conditions are of great importance in fatigue life investigation [36].

Vibration fatigue analysis is carried out for the structures operating in the vicinity of natural frequencies [37,38]. Vibration fatigue analysis differs from traditional fatigue analysis. In vibration fatigue analysis, the modal characteristics and structural dynamics of the structure are considered in dynamic stress simulation [39].

Vibration fatigue is affected by variations in the natural frequency [40] and damping of the structure [41,42]. Thus, fatigue failure can be predicted by utilizing this phenomena of variation in natural frequencies and damping [43]. During fatigue testing, when the frequency of excitation force reaches near the natural frequencies of the structure, resonance effects are raised. The dynamic properties of the structure such as stiffness and damping must be considered in the fatigue life analysis for the increased stress response at resonance [44]. Therefore, instead of using static FEA, a dynamic FEA should be employed to obtain accurate results. For this purpose, stiffness and modal parameters of the system must be known. The stress response in a dynamic FEA model is mainly controlled by the damping of the structure, thereby controlling the durability of the component or structure [45].

Vibration fatigue analysis of steam turbine rotor at resonant conditions was carried out by [46] with different crack lengths in the transverse direction. It was observed that modal parameters changed with crack length ultimately affecting the fatigue life. Moreover, fatigue strength decreased at resonant condition causing failure in less number of cycles compared to non-resonant conditions.

Utilization of lattice structures in various engineering applications has been a popular research area for the past decade. Both weight reduction and improvement in mechanical properties can be accomplished using lattice structures. However, most of the studies focused only on utilization of lattice structures in static components. Utilization of lattice structures in rotating components subjected to vibratory stresses such as turbine rotor blades still lacks experimental investigations. This research gap is addressed in this work by performing vibration fatigue life estimation of octet-truss lattice based blades as a first step to establish their suitability as a potential replacement for conventional solid blades.

## 2. Theoretical Background

The damage in mechanical components is commonly recognized by utilizing the suitable statistical parameters as the variable amplitude loads in the time domain [47]. Consider *N_f_* as a sequence of variable amplitude. The probability density function (PDF), *P_X_* is described as:

(1) $$P_{x\;}\left(N_{f}\right)=\frac{1}{\sqrt{2\pi {\left(SD\right)}^{2}}}e^{-\frac{{\left(N_{f}-\bar{x}\right)}^{2}}{2SD^{2}}}$$

For data with a sample size *N_f_*, the mean $x^{-}$ can be expressed as: [48]

(2) $$x^{-}=\frac{1}{\;Nf}\sum_{j=1}^{n}x_{j}$$

Standard deviation (*SD*) is used to estimate the spread of data around the mean [49]. *SD* is defined as:

(3) $$SD={\left\{\frac{1}{\;\left(Nf-1\right)}\sum_{j=1}^{n}{\left(x_{j}-\bar{x}\right)}^{2}\right\}}^{1/2}$$

In order to statistically evaluate the energy in a signal and explain signal properties, the root mean square (*RMS*) is commonly used approach [49]. The *RMS* value of a variable load *x*, is defined as:

(4) $$RMS={\left\{\frac{1}{\;Nf}\sum_{j=1}^{n}x_{j}{}^{2}\right\}}^{1/2}$$

*Kurtosis* is another important statistical parameter which is used define the non-Gaussian characteristics of a distribution. It is the measure of spikedness of a measured signal. It is computed on the basis of the fourth central moment of a variable data *x*, and is expressed:

(5) $$Kurtosis=\frac{1}{\;Nf{\left(RMS\right)}^{4}}\sum_{j=1}^{n}{\left(x_{j}-\bar{x}\right)}^{4}$$

*Kurtosis* with a value of 3 represents a Gaussian distribution. Higher values of kurtosis (higher than 3) represent the leptokurtic process, whereas values of kurtosis less than 3 represent the platykurtic process. The higher amplitudes in fatigue damage analysis represent high damage. Kurtosis is affected by peaks present in a variable load data [48].

There are various approaches for the estimation of fatigue life in the frequency domain. These approaches are known as PSD cycle counting methods. The narrow-band, Lalanne and Dirlik methods are among the notable approaches in this regard.

In the narrow-band approach, Rayleigh distribution is assumed for the probability of stress peaks. The narrow-band method is only suitable for narrow-band processes. It provides more conservative life estimates when the measured signal is a broad band instead of being a narrow band. This is a major limitation of the narrow-band approach. In this method, the power spectral density (PSD) stress responses are calculated by the expression below [50]:

(6) $$N\left(S\right)=E\left[P\right]\frac{S}{4m_{0}}e^{\frac{S^{2}}{8m_{0}}}$$

where $N\left(S\right)$ represents the number of stress cycles per second and S describes the probability density function of stress range, *m*_0_, *m*_1_, *m*_2_, *m*_4_ are spectral moments, and $E\left[P\right]$ represents the expected number of peaks.

The general expression for estimating the spectral width is given below:

(7) $$\alpha_{i=}\frac{m_{0}}{\sqrt{m_{0}m_{2}}}$$

The parameter *α_i_* has values from 0 to 1. The higher value indicates the narrow width in the frequency domain and vice versa. The expected rate of zero crossing $E\left[0\right]$ and occurrence of peak $E\left[P\right]$ are defined as [51].

(8) $$E\left[0\right]=\sqrt{\frac{m_{2}}{m_{0}}}$$

(9) $$E\left[P\right]=\sqrt{\frac{m_{4}}{m_{2}}}$$

The irregularity factor is given as below:

(10) $$\gamma =\frac{E\left[0\right]}{E\left[P\right]}$$

In the Lalanne approach, the probability density function for a stress range (*S*) is expressed as below [50]:

(11) $$N\left(S\right)=E\left[P\right]\;p\left(S\right)$$

$p\left(S\right)$ can be calculated as follows:

(12) $$p\left(S\right)=\frac{1}{2RMS}\left\{\sqrt{\frac{1-\gamma^{2}}{2\pi }\;}e^{\frac{S^{2}}{8RMS^{2}\left(1-\gamma^{2}\right)}\;}+\frac{S\cdot \gamma }{4RMS}\;e^{\frac{-S^{2}}{8RMS^{2}}\;}\left[1+erf\left(\begin{array}{c} S\cdot \gamma \\ 2RMS\sqrt{2(1-\gamma^{2}} \end{array}\right)\right]\right\}$$

where ‘erf’ is the predicted rain flow count per second and is given as:

(13) $$\mathrm{erf}\left(S\right)=\frac{2}{\sqrt{\pi }}\int_{0}^{x}e^{-t^{2}}dt$$

According to this approach, the probability density function (PDF) changes according to predicted rain flow count per second (erf).

The Dirlik approach is another commonly used method for the estimation of PDF of a stress range. Four moments of areas of PSD are used in this approach. The loading peaks are expressed as [52]:

(14) $$p\left(S\right)=\frac{\frac{D1}{Q}e^{\frac{-Z}{Q}}+\frac{ZD_{2}}{R^{2}}\;e^{\frac{-Z^{2}}{2R^{2}}}+D_{3}Ze^{\frac{-Z^{2}}{2}}}{2\sqrt{m_{0}}}$$

The function of four spectral moments ($m_{0},m_{1},m_{2},\;m_{4}$) are represented as.

(15) $$D_{1}=\frac{2\left(X_{m}-\gamma^{2}\right)}{1+\gamma^{2}}$$

(16) $$D_{2}=\frac{\left(1-\gamma -D_{1}+D_{1}{}^{2}\right)}{1+R}$$

(17) $$D_{3}=1-D_{1}-D_{2}$$

(18) $$R=\frac{\left(\gamma -x_{m}-D_{1}{}^{2}\right)}{1-\gamma -D_{1}+D_{1}{}^{2}}$$

where

(19) $$x_{m}=\frac{m_{1}}{m_{0}}\sqrt{\frac{m_{2}}{m_{4}}}$$

The Dirlik approach can be applied to various types of loading. Both the Lalanne and Dirlik methods are considered to be robust and reliable for obtaining the accurate cycle count for both narrow-band and wide-band signals [53]. Loading in the form of vibration acceleration must be converted into stress using some stress criterion.

## 3. Materials and Methods

**Figure 1:**
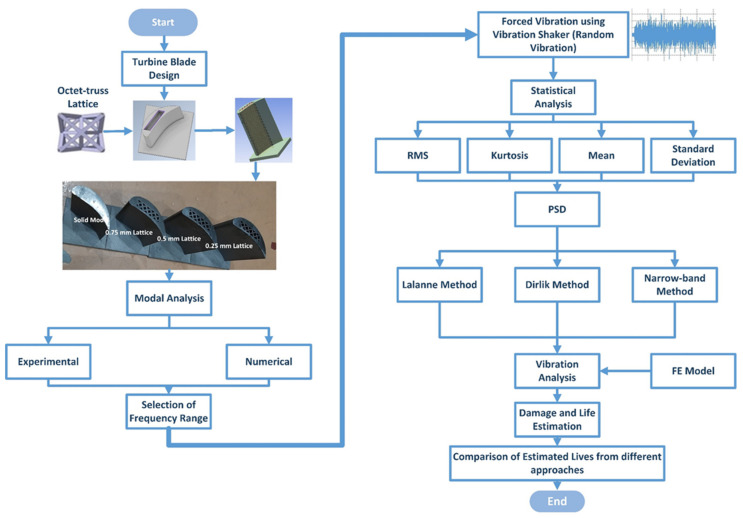
Research framework.

This work aims to estimate the vibration-based fatigue life of octet-truss lattice-based turbine rotor blades. The framework of this research is illustrated in Figure 1. In the first step, turbine blades were designed and manufactured via additive manufacturing. After manufacturing, modal analysis was performed on the blades to determine their natural frequencies and associated mode shapes. Both experimental and numerical techniques were utilized for modal analysis. After getting modal parameters, forced response analysis was performed on each blade by applying random vibration using a vibration shaker. The input signal and the response were measured using uniaxial and triaxial accelerometers, respectively. After data acquisition, statistical analysis was performed to obtain the power spectral density of each signal. The data were further processed in nCode^®^ Designlife^®^ to obtain the cycle counts using PSD cycle counters. Three approaches, namely, Lalanne, Dirlik and narrow band, are utilized for the cycle counting. After cycle counting, vibration analysis in nCode^®^ Designlife^®^ is performed with PSD cycle counts and finite element model (FEM) as inputs. From vibration analysis, both damage and life cycle estimates are obtained.

### 3.1. Blade Design and Manufacturing

**Figure 2:**
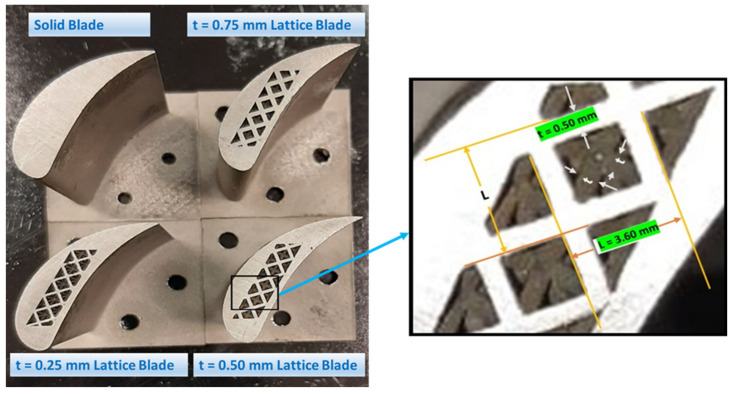
Turbine blades used in this study.

In this work, four turbine blades were designed and manufactured with Inconel 718 as blade material. Modern gas turbines operate at high temperatures (up to 1600 °C inlet temperature) to achieve higher efficiencies. This require turbine blade materials with good thermal and mechanical properties at elevated temperatures. Compared to conventional cast (CC) blades, the directional solidified (DS) and single crystal (SC) blades exhibit better thermal and mechanical characteristics at high temperatures as discussed by [54]. However, the material selection in this study was made based on the limitations of available additive manufacturing facility. The advancements in the field of additive manufacturing can overcome these limitation to select the modern turbines’ blade materials such as Mar M247, CM 247 LC, CMSX 2 and PWA 1484. One blade was complete solid whereas three blades were octet-truss lattice based. A solid blade was designed in Autodesk Inventor 2021. NACA 23012, a commonly used airfoil for gas turbine blades, was selected for profile design of the blades. The octet-truss lattice structure was selected for blade infill given its good mechanical properties [55], which can be ascribed to its unique shape. The lattice part was designed in CATIA V5R21. For lattice blades, the unit cell size was fixed at 3.60 × 3.60 mm whereas the strut thickness (t) was variable. Given that the lattice structures are porous in nature, the blade cannot be completely comprise of a lattice structure due to its operational requirements. Therefore, lattice part should be placed inside a cavity created in the solid blade. Three lattice blades were designed by filling the cavity inside the blade with a strut thickness of 0.75, 0.50 and 0.25 mm, as shown in Figure 2. In this study, only structural loadings are considered in the form of random vibrations. Therefore, the lattice-based blades were designed by keeping in view the structural loading conditions alone. In order to incorporate the thermal and cooling effects in the analysis, design changes such as use of conformal lattice can be used to maximize the lattice portion in blade especially near the leading edge, which will be the focus of an upcoming numerical investigation. Additive manufacturing was carried out using selective laser melting (SLM) technique on Renishaw RenAM 500E machine to manufacture all four blade samples [55]. The SLM parameters are listed in Table 1.

Due to variation in strut thickness (t), each lattice blade has a different weight, thereby resulting in different levels of weight reduction achieved compared to the solid blade as described in Table 2.

### 3.2. Modal Analysis

For the investigation of vibration fatigue characteristics, it is mandatory to understand the structural dynamics of the blades which requires to determine the modal parameters. In this work, modal analysis is performed numerically as well as experimentally. Numerical modal analysis was performed in ANSYS 2022 R1. For mode extraction, the Block Lanczos method was used due to its high convergence rate. A tetrahedron mesh type was used with a mesh size of 0.125 mm. A Fixed support as boundary condition was applied at the bottom base of the blade. The physical and mechanical properties used for modal analysis are listed in Table 3.

**Figure 3:**
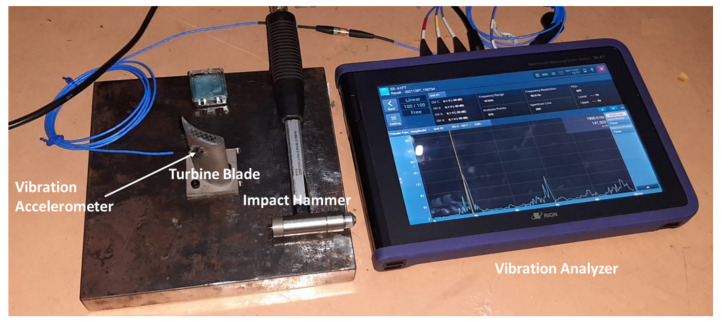
Experimental modal analysis test setup.

An impact testing (ISO 7626-5) technique was employed for experimental modal analysis. Turbine blades were mounted on a rigid mild steel base plate. A triaxle piezoelectric accelerometer (PCB 356A01) was mounted on the blade using adhesive. The blade was excited by a modally tuned impact hammer (PCB 086C03), and the response was measured using vibration analyzer (RIONOTE SA-A1). The measured response/input (g/N) was plotted against the frequency range to obtain the natural frequencies of the blades. A frequency range of 0–7 KHz was used with a sampling rate of 25.6 kilo samples per second. The experimental setup for modal analysis is shown in Figure 3.

### 3.3. Forced Vibration Response

**Figure 4:**
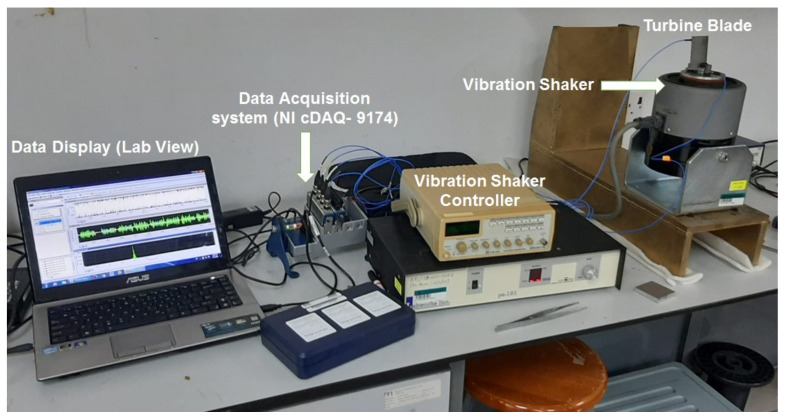
Setup for vibration fatigue testing.

In modal analysis, the free vibration response of the system is measured. However, for vibration fatigue life estimation, the blades must be subjected to continuous excitation using a vibration shaker. The experimental setup for forced vibration analysis is illustrated in Figure 4.

**Figure 5:**
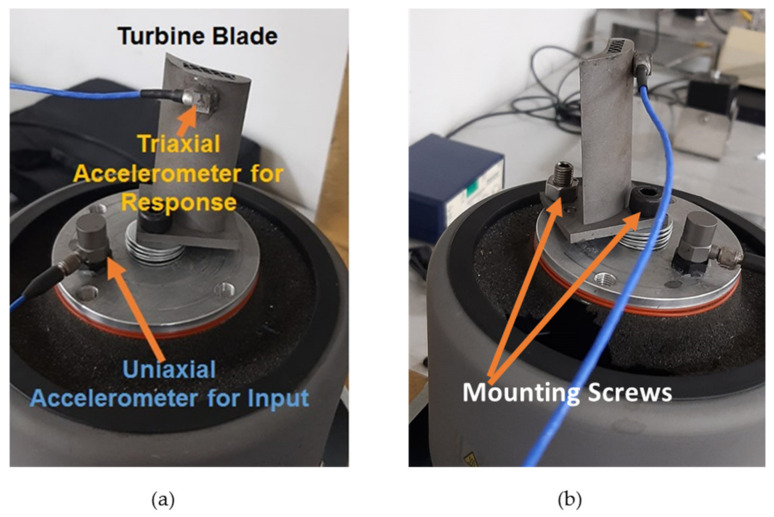
Blade mounting on the vibration shaker: (**a**) accelerometer mounting; (**b**) illustration of mounting screws.

For vibration fatigue analysis, the blades were mounted on the vibration shaker (Labworks ET-139) using mounting screws. The rigidity of the mounting must be ensured for accurate results. Each blade was subjected to random vibration with similar RMS value of 2 g to ensure the same loading conditions for each blade. The input vibration signal was controlled and adjusted using the vibration shaker controller. The response was measured in the time domain in terms of vibration acceleration. One triaxial accelerometer was used to measure the blade response, while a uniaxial accelerometer was used to measure the input excitation signal as illustrated in Figure 5.

### 3.4. Vibration Fatigue Analysis

**Figure 6:**
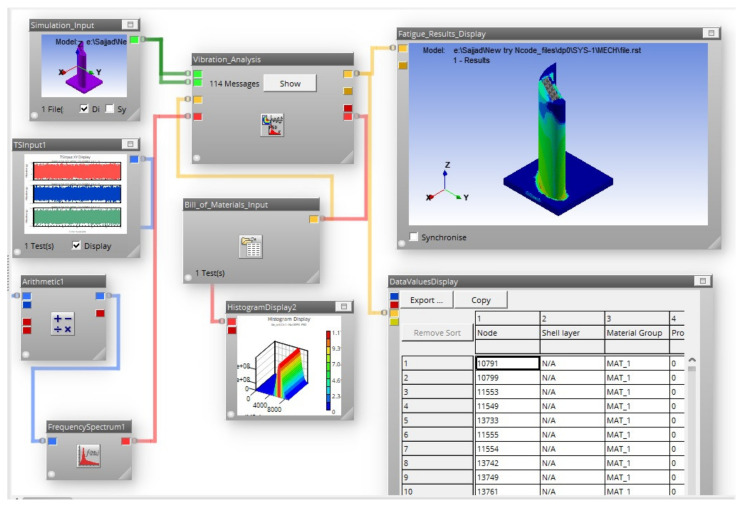
nCode^®^ Designlife^®^ analysis scheme used for vibration fatigue analysis.

After acquisition of vibration acceleration signal in the time domain for each blade, vibration-based fatigue analysis was performed using nCode^®^ Designlife^®^. The acquired signal was processed to obtain the statistical parameters of the signal such as RMS, kurtosis, mean and standard deviation to determine the type of the data. In next step, conversion of the time domain data to power spectral density (PSD) is carried out. After PSD, the cycle counting is carried out using three commonly used approaches named Lalanne, Dirlik and narrow band. Modal analysis and harmonic analyses were performed on each blade to obtain the input FE models for vibration fatigue analysis. In harmonic analysis, unit vibration acceleration of 1 g (9.81 m/s^2^) was applied to obtain the stress data. Vibration analysis was performed in nCode^®^ Designlife^®^ by using the results of numerical modal and harmonic analysis (as FE model input), applied with the vibration load in terms of PSD of the actual measured acceleration for each blade. There are various stress criterion available in nCode^®^ Designlife^®^ vibration analysis such as absolute maximum principal and critical plane. The absolute maximum principal stress criterion is used in this work. Results were obtained in the form of damage and fatigue life cycles from vibration analysis. The schematic of turbine blade fatigue life analysis in nCode^®^ Designlife^®^ is illustrated in Figure 6. The cyclic properties used for the vibration fatigue life analysis are listed in Table 4.

## 4. Results and Discussion

Both free vibration and forced vibration response were investigated in this study to determine the suitability of octet-truss lattice structure utilization in turbine blade applications. Free vibration analysis results were obtained in the form of natural frequencies and mode shapes, while forced vibration analysis was performed to investigate the vibration fatigue characteristics of the turbine blades subjected to random vibration loading.

### 4.1. Modal Analysis Results

**Figure 7:**
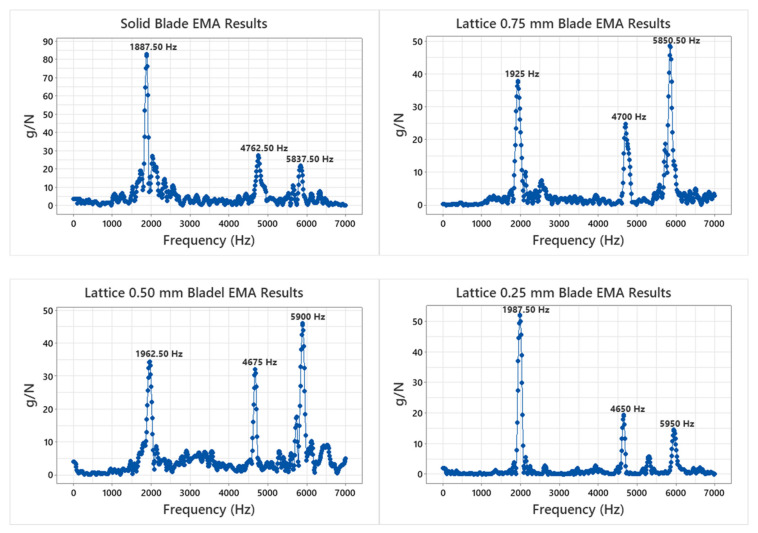
Experimental modal analysis results.

Experimental and numerical modal analysis was performed to obtain the resonant natural frequencies and mode shapes for turbine blades. Figure 7 shows the experimental modal analysis results for each turbine blade. Each distinct peak corresponds to a natural frequency. For a selected frequency range of 7000 Hz, each turbine blade has three natural frequencies, with lattice blades having higher natural frequencies compared to the solid blade. Results indicate that of all blades, the solid blade has exhibited the lowest natural frequency (1887.50 Hz), while the 0.25 mm lattice blade exhibited the highest natural frequency (1987.50 Hz) at this mode, which is 5.29% higher compared to that of the solid blade. This indicates that decreasing the diameter of the octet-truss struts results in extension of the first natural frequency. This is a significant advantage of using octet-truss lattice structures as along with weight reduction, enhancement of natural frequency is also achieved, which can increase the operating range of turbine for rigid mode rotor operation. A similar trend is observed at the third mode as well. However, at the second mode, this trend is inversed, i.e., the solid blade has a maximum natural frequency (4762.5 Hz), while the 0.25 mm lattice blade has the lowest (4650 Hz) of all blades.

In order to validate the experimental modal analysis results, numerical modal analysis was also performed. A maximum difference of 3.9% (at the first mode for the solid blade) was observed between experimental and numerical analyses results.

**Figure 8:**
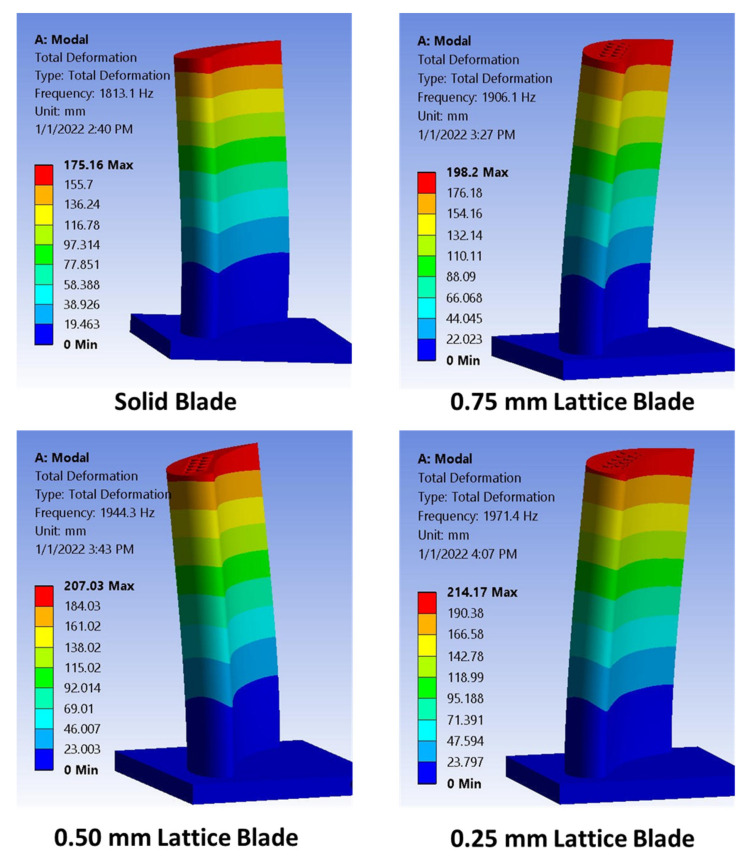
Mode shapes of turbine blades at the first natural frequency.

Mode shapes for turbine blades at the first natural frequency are shown in Figure 8. For all turbine blades, the first mode is the flap wise bending mode. Based on modal analysis results, the excitation frequencies are selected for each turbine blade to achieve resonant condition. Only the first natural frequency is selected in this study for forced vibration analysis on a vibration shaker.

### 4.2. Forced Vibration Analysis Results

**Figure 9:**
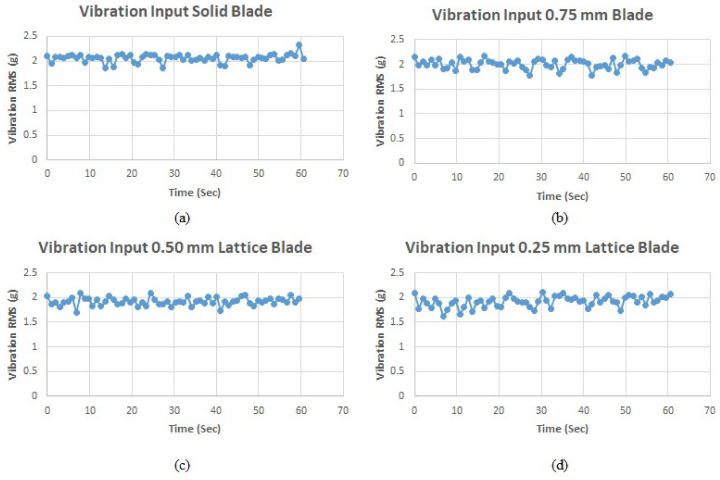
Input vibration signal for blades: (**a**) the solid blade; (**b**) the 0.75 mm lattice blade; (**c**) the 0.50 mm lattice blade; (**d**) the 0.25 mm lattice blade.

**Figure 10:**
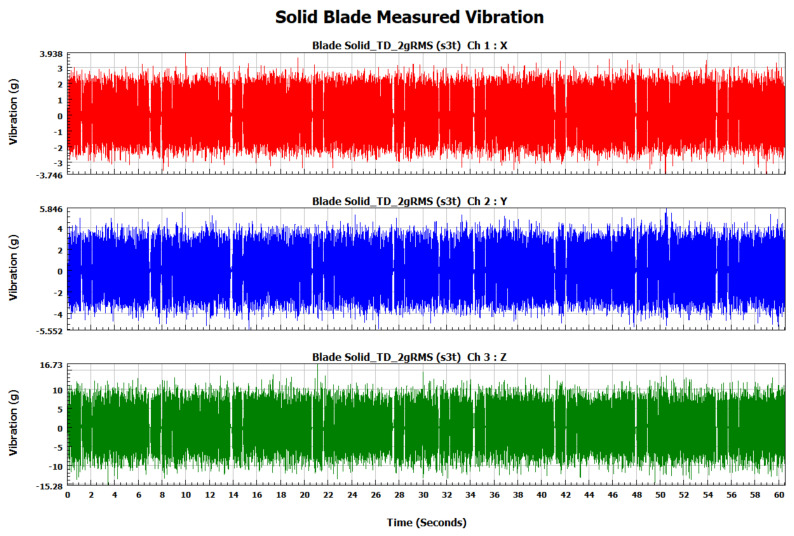
Solid blade measured vibration signal in the time domain.

**Figure 11:**
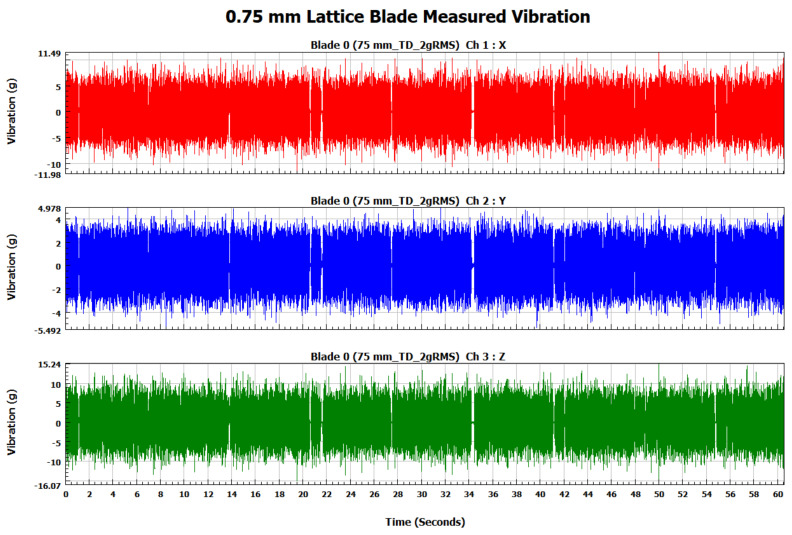
The 0.75 mm lattice blade measured vibration signal in the time domain.

**Figure 12:**
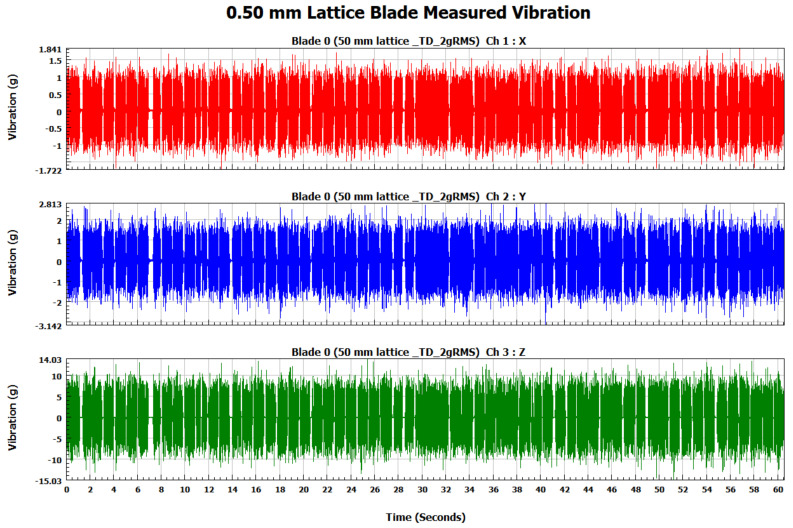
The 0.50 mm lattice blade measured vibration signal in the time domain.

**Figure 13:**
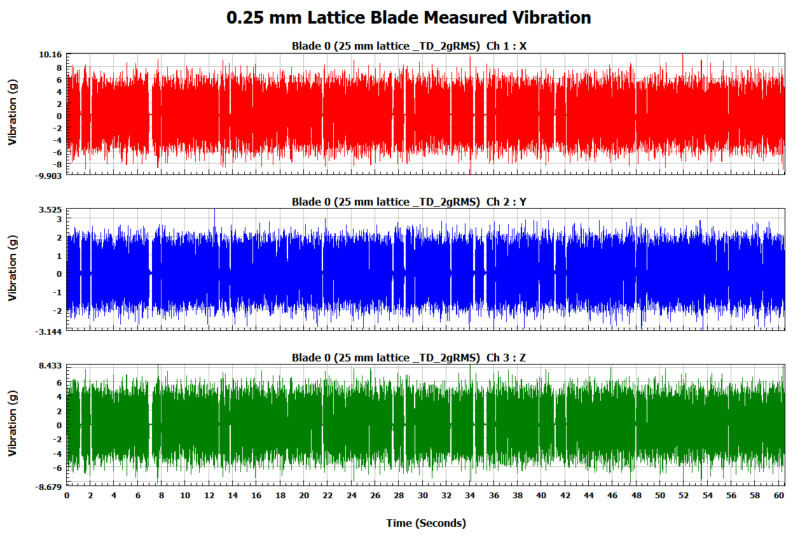
The 0.25 mm lattice blade measured vibration signal in the time domain.

The forced vibration analysis was performed to investigate the response of the turbine blades when subjected to vibration loads of random nature similar to the ones experienced during their operational life. The response of each blade was measured in terms of vibration acceleration in the X, Y, and Z directions for each blade. In order to ensure similar loading conditions for each blade, the applied vibration signal was controlled by its RMS value using a vibration shaker. As shown in Figure 9, the RMS of the input signal for each blade is 2 g (19.62 m/s^2^). The measured response signals in terms of vibration acceleration for the solid, 0.75, 0.50, and 0.25 mm lattice blades in the time domain are shown in Figure 10, Figure 11, Figure 12 and Figure 13, respectively.

The statistical characteristics of these measured signals are presented in Table 5. The kurtosis for all the measured signals is above 3.0, which indicates that all the measured signals are non-Gaussian and non-stationary in nature. This indicates the measured signal is identical to the actual conditions of the blade as vibration signals experienced during operational life are usually non-Gaussian [37].

The measured signals are then processed in nCode^®^ Designlife^®^ to obtain the power spectral density (PSD) and cycle count for each signal. For this purpose, the frequency domain approaches, namely, Lalanne, Dirlik and narrow band, are used. Due to different governing equations, each approach yields different cycle count results.

**Figure 14:**
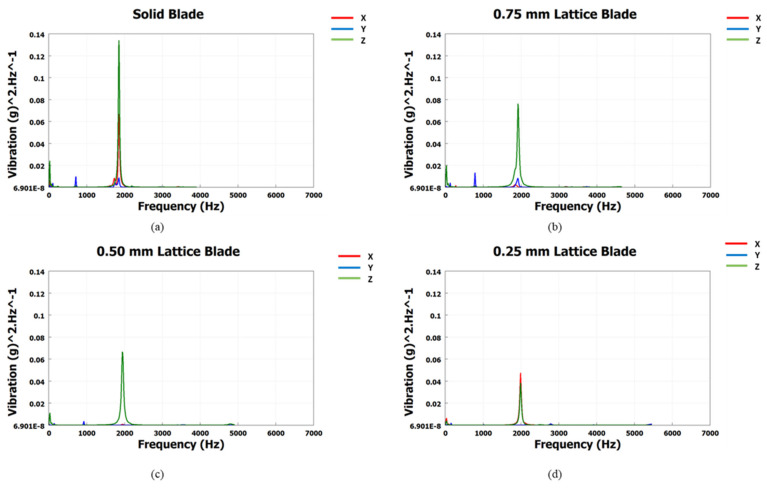
Response PSD results of turbine blades: (**a**) the solid blade; (**b**) the 0.75 mm lattice blade; (**c**) the 0.50 mm lattice blade; (**d**) the 0.25 mm lattice blade.

The PSD results for each blade in the X, Y, and Z directions are shown in Figure 14. The PSD results represent the vibration energy of each signal in terms of g^2^/Hz. The vibration energy is maximum at the natural frequencies.

**Figure 15:**
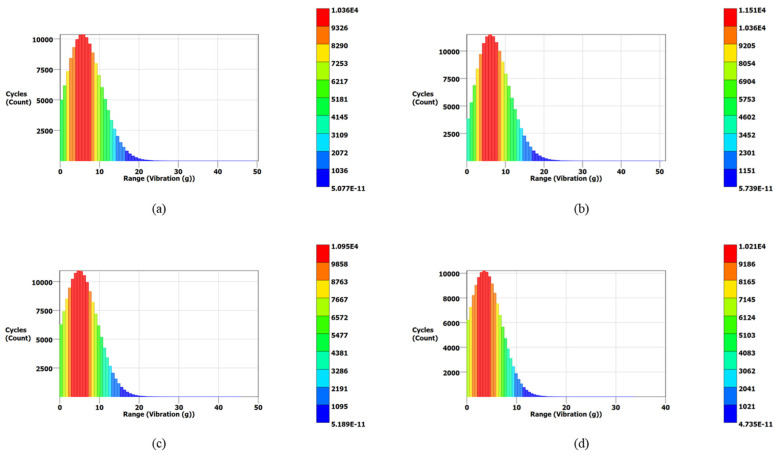
PDF results for turbine blades using the Lalanne approach: (**a**) the solid blade; (**b**) the 0.75 mm lattice blade; (**c**) the 0.50 mm lattice blade; (**d**) the 0.25 mm lattice blade.

**Figure 16:**
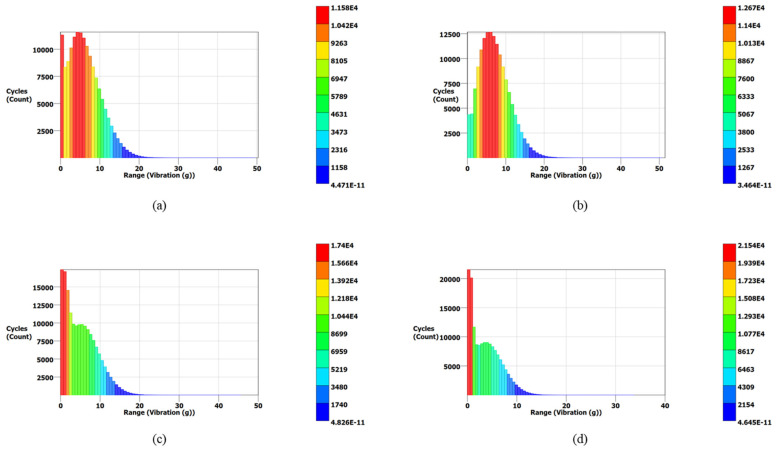
PDF results for turbine blades using the Dirlik approach: (**a**) the solid blade; (**b**) the 0.75 mm lattice blade; (**c**) the 0.50 mm lattice blade; (**d**) the 0.25 mm lattice blade.

**Figure 17:**
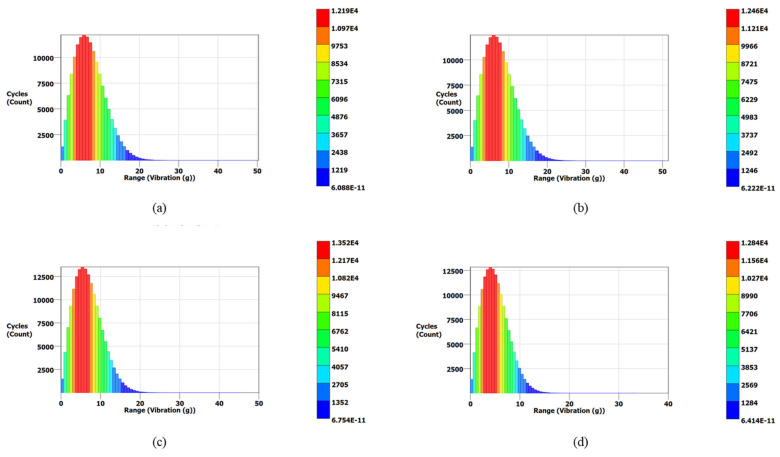
PDF results for turbine blades using the narrow-band approach: (**a**) the solid blade; (**b**) the 0.75 mm lattice blade; (**c**) the 0.50 mm lattice blade; (**d**) the 0.25 mm lattice blade.

Figure 14 also validates the modal analysis results for the first natural frequency of each blade. From Figure 14, the first natural frequencies of the solid, 0.75, 0.50, and 0.25 mm lattice blades are observed as 1837, 1912, 1940, and 1995 Hz, respectively, identical to those obtained from modal analysis. The maximum vibration energies of 0.13, 0.08, 0.07, and 0.05 g^2^/Hz are observed for the solid, 0.75, 0.50, and 0.25 mm lattice blades, respectively. These PSD signals were then processed using three different frequency domain approaches for cycle counting to obtain the equivalent decomposed simple reversal cycles for each case. For each blade, the vibration signal maximum range (g) and corresponding occurrence in terms of cycles are obtained. The cycle counting results using the Lalanne, Dirlik, and narrow-band approaches for the measured responses are illustrated in Figure 15, Figure 16 and Figure 17, respectively, while a summary of the results is presented in Table 6.

In the Lalanne approach, the probability density function (PDF) of peaks is assumed to be the simple sum of Gaussian and Rayleigh distributions [58], while the Dirlik approach assumes the PDF of peaks is a combination two Rayleigh densities and an exponential. Due to this assumption, the cycle counting distribution in the Dirlik approach decays faster compared to that of the Lalanne method, as shown in Figure 16. In the narrow-band approach, amplitude distribution in Rayleigh form is assumed for cycle counting. This method overestimates the fatigue damage for wide-band signals; thus, it is more conservative for wide-band signals [59].

**Figure 18:**
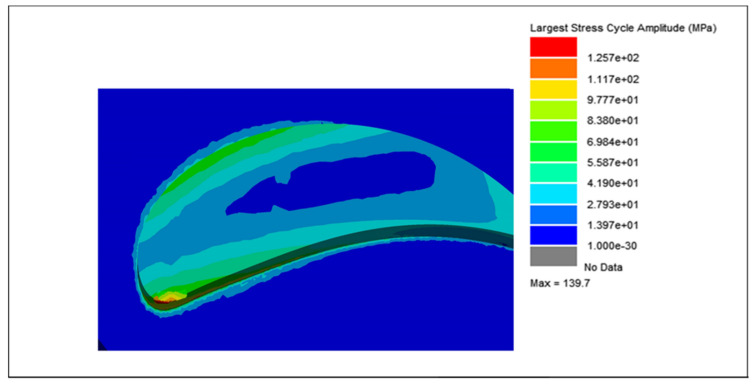
Largest stress cycle amplitude for the solid blade.

**Figure 19:**
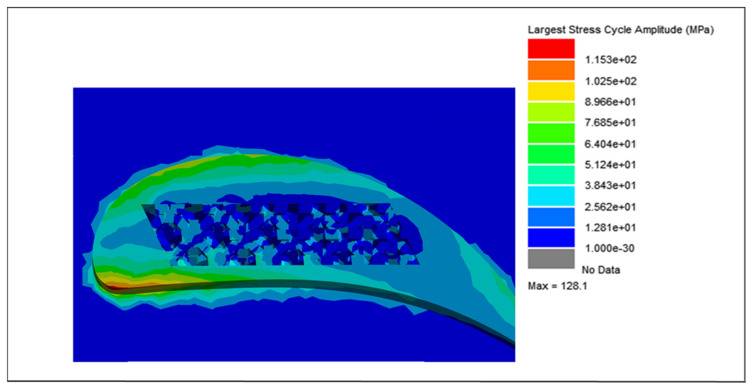
Largest stress cycle amplitude for the 0.75 mm lattice blade.

**Figure 20:**
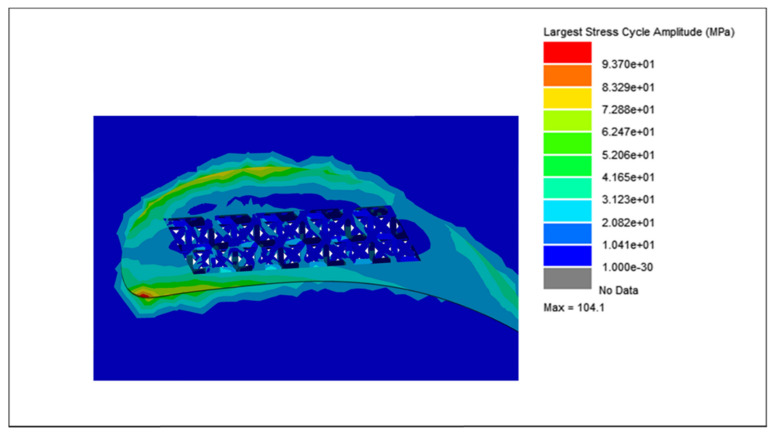
Largest stress cycle amplitude for the 0.50 mm lattice blade.

**Figure 21:**
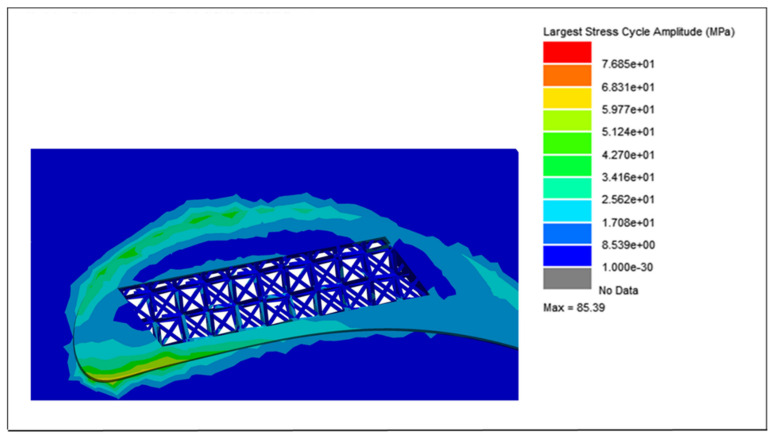
Largest stress cycle amplitude for the 0.25 mm lattice blade.

The cycle counting data, integrated with the modal and harmonic analyses of the blades, were further processed using the vibration analysis tool in nCode^®^ Designlife^®^ for fatigue damage and life estimations of each blade. Due to the variations in weight and lattice unit cell strut thickness, the largest stress cycle amplitude for each blade is different. The largest stress cycle amplitudes for each blade are shown in Figure 18, Figure 19, Figure 20 and Figure 21, respectively.

For the solid blade, the largest stress cycle observed was 139.7 MPa, while the largest stress cycles for the 0.75, 0.50 and 0.25 mm lattice blades observed were 128.10, 104.1, and 85.3 MPa, respectively. This difference in largest stress cycles resulted in different fatigue lives for each blade.

Finally, the fatigue damage and fatigue life results obtained from vibration analysis for the solid, 0.75, 0.50 and 0.25 mm lattice blades are listed in Table 7, Table 8, Table 9 and Table 10, respectively. For all three cycle counting approaches, the results indicate that lattice-based blades exhibit less damage compared to the solid blade, resulting in better fatigue lives. Using the Lalanne approach, the 0.25 mm lattice blade exhibits the least damage of all the blades, which is 2.08 × 10^−13^ cycles, which implies a fatigue life of 4.81 × 10^12^ blocks to failure. The solid blade exhibited a fatigue life of 2.64 × 10^9^ blocks to failure, which was the lowest of all four blades. Using the Lalanne approach, the estimated fatigue lives were 35-, 246-, and 1822-fold higher for the 0.75, 0.50, and 0.25 mm lattice blades, respectively, compared to that of the solid blade.

The damage results listed in Table 7, Table 8, Table 9 and Table 10 obtained using the Dirlik approach differ from those obtained using the Lalanne approach of cycle counting. The respective largest stress cycles for each blade were the same as those in the Lalanne approach but the degree of damage is different due to the difference in cycle counting methods for the Lalanne and Dirlik approaches. The Dirlik approach resulted in less damage compared to the Lalanne approach for all blades, hence resulting in higher fatigue lives. This trend was also reported in previous works such as [60]. The maximum damage was observed in the solid blade followed by the 0.75, 0.50, and 0.25 mm lattice blades, respectively, showing a similar trend to that observed using the Lalanne approach. For the solid blade, the fatigue life observed was 3.85 × 10^9^ blocks to failure, which is the minimum of all blades. The 0.75, 0.50, 0.25 mm lattice blades exhibited a 35-, 258-, and 1802-fold higher fatigue life compared to that of the solid blade.

The results of fatigue damage estimations using the narrow-band approach also indicate a similar trend, where the highest damage is observed for the solid blade and the lowest for the 0.25 mm lattice blade of all blades using the Lalanne and Dirlik approaches. However, the magnitude of damage differs for the Lalanne and Dirlik approaches. Due to its conservative nature, for wide-band signals, the narrow-band approach resulted in the highest damage of all approaches for respective blades. Using the narrow-band approach, the fatigue life obtained for the solid blade was 1.94 × 10^9^ blocks to failure. The 0.75, 0.50, and 0.25 mm lattice blades exhibited a 35-, 260-, and 1819-fold higher fatigue life compared to that of the solid blade using the narrow-band approach.

It is evident from the fatigue life results that, compared to the solid blade, lattice blades have higher life cycles and the fatigue life improves with a reduction in the strut thickness of the lattice unit cells. Of the three frequency domain approaches used in this study, the Dirlik approach resulted in the highest fatigue life followed by the Lalanne and the narrow-band approaches, which is in agreement with the results of previous studies such as [52].

## 5. Conclusions

Free and forced vibration analyses of octet-truss lattice infill turbine rotor blades were performed in this study to investigate their vibration fatigue characteristics in comparison to that of the conventional solid blade. Lattice structures are being utilized in various engineering applications due to their ability to reduce the weight of a component in a given design space and better mechanical properties. Based on the results, the following conclusions are drawn:

1. Using octet-truss lattice structures with variable strut thickness, a weight reduction of 15.58% to 24.91% compared to the solid blade was achieved.
2. The natural frequencies of lattice infilled blades were found to be higher than those of solid blades at the first and third modes.
3. For vibration fatigue analysis, three frequency domain fatigue approaches were utilized. The results indicate that the Dirlik approach exhibited the highest fatigue lives, while the narrow-band approach resulted in the lowest fatigue lives for the respective blades.
4. Lattice-based blades have better fatigue lives compared to the solid blade; the 0.25 mm lattice blade, which is the lightest of all blades, exhibited a the least damage of all four blades followed by the 0.50 and 0.75 mm lattice blades, respectively.

Therefore, it can be concluded that octet-truss lattice infill blades exhibit better vibration fatigue characteristics compared to solid blades, making them a potential replacement for conventional solid blades, enhancing durability. However, further studies are required to investigate the cooling effectiveness and effect of combined vibration and thermal loads on the fatigue life of the blades in order to fully establish the utility of octet-truss lattice infill blades in turbine rotors.

## Figures and Tables

**Table 1:** SLM parameters for additive manufacturing of blades.

Parameter	Description
Laser power	200 W
Laser speed	875 mm/s
Layer thickness	60 μm
Hatching distance	90 μm
Energy density	42.32 J/mm^3^

**Table 2:** Weight of turbine blades used in this study.

Blade Description	Weight (kg)	Weight Reduction from Complete Solid (%)
Complete Solid	0.185	0
0.75 mm Lattice	0.156	15.58
0.50 mm Lattice	0.147	20.78
0.25 mm lattice	0.139	24.91

**Table 3:** Properties of Inconel 718 used in the modal analysis [56].

Property	Description
Density	8190 Kg/m^3^
Young’s Modulus Poisson’s ratio	200 GPa 0.3
Ultimate Tensile Strength Yield Tensile Strength	1375 MPa 1100 MPa
Bulk Modulus	137 GPa
Shear Modulus	63.46 MPa

**Table 4:** Cyclic properties of Inconel 718 [57].

Property	Description
Cyclic strength coefficient	776.21 MPa
Fatigue strength coefficient	725.52 MPa
Fatigue strength exponent Fatigue ductility coefficient	−0.066 0.990
Fatigue ductility exponent	−0.701
Cyclic strain hardening exponent	0.0942

**Table 5:** Statistical analysis of measured vibration signals.

Parameter (g)	Solid Blade			0.75 mm Lattice Blade			0.50 mm Lattice Blade			0.25 mm Lattice Blade		
	X	Y	Z	X	Y	Z	X	Y	Z	X	Y	Z
RM	0.739	1.15	2.06	2.06	1.09	2.13	0.33	0.54	2.12	2.01	0.61	1.77
Kurtosis	3.46	3.37	3.66	3.41	3.21	3.33	3.91	3.76	3.91	3.43	3.58	3.46
Mean	0.016	0.006	0.024	0.016	0.008	0.024	0.015	0.007	0.023	0.015	0.008	0.022
Standard Deviation	0.739	1.15	2.06	2.06	1.09	2.13	0.33	0.54	2.12	2.01	0.61	1.76

**Table 6:** PSD cycle count for each blade measured response.

Cycle Counter	Solid Blade		0.75 mm Lattice Blade		0.50 mm Lattice Blade		0.25 mm Lattice Blade	
	Range (g)	Cycle Count	Range (g)	Cycle Count	Range (g)	Cycle Count	Range (g)	Cycle Count
Lalanne	50.34	5.07 × 10^−11^	51.50	5.73 × 10^−11^	46.36	5.18 × 10^−11^	34.28	4.73 × 10^−11^
Dirlik	50.34	4.47 × 10^−11^	51.50	3.46 × 10^−11^	46.36	4.82 × 10^−11^	34.28	4.64 × 10^−11^
Narrow Band	50.34	6.08 × 10^−11^	51.50	6.22 × 10^−11^	46.36	6.75 × 10^−11^	34.28	6.41 × 10^−11^

**Table 7:** Fatigue life results for the solid blade.

Cycle Counter	Largest Stress Cycle Amplitude (MPa)	Fatigue Damage	Fatigue Life (Blocks to Failure)
Lalanne	139.7	3.79 × 10^−10^	2.64 × 10^9^
Dirlik	139.7	2.60 × 10^−10^	3.85 × 10^9^
Narrow Band	139.7	5.15 × 10^−10^	1.94 × 10^9^

**Table 8:** Fatigue life results for the 0.75 mm lattice blade.

Cycle Counter	Largest Stress Cycle Amplitude (MPa)	Fatigue Damage	Fatigue Life (Blocks to Failure)
Lalanne	128.1	1.06 × 10^−11^	9.39 × 10^10^
Dirlik	128.1	7.41 × 10^−12^	1.35 × 10^11^
Narrow Band	128.1	1.45 × 10^−11^	6.90 × 10^10^

**Table 9:** Fatigue life results for the 0.50 mm lattice blade.

Cycle Counter	Largest Stress Cycle Amplitude (MPa)	Fatigue Damage	Fatigue Life (Blocks to Failure)
Lalanne	104.1	1.54 × 10^−12^	6.49 × 10^11^
Dirlik	104.1	1.01 × 10^−12^	9.94 × 10^11^
Narrow Band	104.1	1.98 × 10^−12^	5.04 × 10^11^

**Table 10:** Fatigue life results for the 0.25 mm lattice blade.

Cycle Counter	Largest Stress Cycle Amplitude (MPa)	Fatigue Damage	Fatigue Life (Blocks to Failure)
Lalanne	85.3	2.08 × 10^−13^	4.81 × 10^12^
Dirlik	85.3	1.44 × 10^−13^	6.94 × 10^12^
Narrow Band	85.3	2.83 × 10^−13^	3.53 × 10^12^

## Data Availability

Data are available upon request from the corresponding author. These data are not commercially available due to privacy issues.

## Nomenclature

$m_{i}$	The ith spectral moment
$\alpha_{i\;}$	Spectral width
$E\left[P\right]$	Expected occurrence of peak
$E\left[0\right]$	Expected rate of zero crossing
γ	Irregularity factor
$P_{x}$	Probability density function
x	Variable load
$\bar{x}$	Mean
*N_f_*	Data sample size
$N\left(S\right)$	Number of stress cycles expected per second
S	Probability density function of stress range
erf	Predicted rain flow count per second
$D_{i}$	ith function of spectral moments
R	Function of spectral moments
